# Sea Squirt-Derived Peptide WLP Mitigates OKA-Induced Alzheimer’s Disease-like Phenotypes in Human Cerebral Organoid

**DOI:** 10.3390/antiox14050553

**Published:** 2025-05-07

**Authors:** Qiqi Chen, Zhiqiu Wang, Wei Guo, Aiqin Xue, Guohui Bian, Xinhua Guo, Shiya Lu, Pinli Zeng, Hao Li, Xizhi Zhu, Yan Huang, Xiaobo Cen, Qian Bu

**Affiliations:** 1 Molecular Toxicology Key Laboratory of Sichuan Provincial Education Office, Institute of Systems Epidemiology, West China School of Public Health and West China Fourth Hospital, Sichuan University, Chengdu 610041, China; 2022224040064@stu.scu.edu.cn (Q.C.); 2024224040077@stu.scu.edu.cn (Z.W.); guoxinhua@stu.scu.edu.cn (X.G.); 2022224040065@stu.scu.edu.cn (S.L.); 2023224045168@stu.scu.edu.cn (P.Z.); 2024224045064@stu.scu.edu.cn (H.L.); 2024324040014@stu.scu.edu.cn (Y.H.); 2 National Chengdu Center for Safety Evaluation of Drugs, State Key Laboratory of Biotherapy, Collaborative Innovation Center of Biotherapy, West China Hospital, West China Medical School, Sichuan University, Chengdu 610041, China; wguo@glpcd.com (W.G.); aiqinxue@glpcd.com (A.X.); guohuibian@glpcd.com (G.B.); zxzqyc0909@163.com (X.Z.); xbcen@scu.edu.cn (X.C.)

**Keywords:** food-derived peptide, Alzheimer’s disease, okadaic acid, cerebral organoids

## Abstract

Alzheimer’s disease (AD), a prevalent neurodegenerative disorder in the elderly, poses significant humanistic and economic burdens worldwide. Previously, we identified Trp-Leu-Pro (WLP), a novel antioxidant peptide derived from the sea squirt (*Halocynthia roretzi*); however, its effects on AD remained unexplored. In this study, we developed a rapid and efficient method to generate AD cerebral organoids with consistent quality using okadaic acid (OKA) exposure. This study aimed to evaluate the protective effects of WLP on OKA-induced AD pathology in cerebral organoids and elucidate its underlying mechanisms. Our results demonstrated that cerebral organoids exposed to 25 nM OKA successfully recapitulated hallmark AD pathologies, including amyloid-beta (Aβ) plaque deposits, neurofibrillary tangles (NFTs) formed by hyperphosphorylated tau proteins, and neuronal loss. WLP treatment significantly enhanced cell viability, increased the proportion of neuronal progenitor cells, and reduced Aβ plaques and NFTs in OKA-induced cerebral organoids. Furthermore, transcriptomic analysis revealed that the neuroprotective effects of WLP are primarily mediated through the regulation of synapse-related and oxidative stress pathways. These findings highlight the potential of WLP as a promising nutraceutical candidate for AD prevention.

## 1. Introduction

Alzheimer’s disease (AD) is a neurodegenerative disorder characterized by progressive cognitive decline and memory impairment, positioning it as one of the foremost causes of dementia globally [1]. The increasing prevalence of AD, particularly with the aging population, presents significant social and economic burdens [2]. The primary pathological features of AD include the accumulation of amyloid-β (Aβ) plaques and hyperphosphorylation of tau proteins, which result in synaptic dysfunction, neurodegeneration, and cognitive decline. Excessive Aβ accumulation triggers neurotoxicity, leading to oxidative stress, which plays a pivotal role in the progression of AD [3,4]. Given the limitations of current pharmacological treatments, there is growing interest in dietary and lifestyle interventions as potential strategies to prevent or delay the onset of AD [5]. Recent research highlights the potential of antioxidant peptides, particularly those derived from functional foods, to mitigate oxidative stress and its associated pathological effects in AD [6]. These peptides offer a promising therapeutic approach by targeting oxidative stress, a key mechanism in AD, and align with a preventive, health-oriented model that focuses on lifestyle changes rather than relying solely on pharmacological treatments [6].

Food-derived bioactive peptides, recognized as promising candidates in nutraceutical innovation, possess substantial potential for preventing various diseases [7]. These peptides have gained increasing attention due to their abundant availability, ease of preparation, environmental sustainability, stability, and minimal side effects [8]. Notably, certain food-derived peptides have demonstrated significant health benefits, particularly their neuroprotective effects [9,10]. Neuroprotective peptides have been shown to inhibit apoptosis, enhance acetylcholine activity, and reduce oxidative stress [11]. For instance, our previous study identified Pro Gly-Cys-Pro-Ser-Thr (PGCPST), a peanut-derived peptide, which ameliorated 6-OHDA-induced apoptosis in PC12 cells through pathways related to sphingolipid metabolism [12]. Furthermore, specific food-derived peptides exhibit protective effects against AD [6]. For example, Leu-Asp-Tyr-Glu (TPM), a maize-derived peptide, was reported to protect *Caenorhabditis elegans* from Aβ-induced damage by attenuating Aβ aggregation [13]. Similarly, the walnut-derived peptide Thr-Trp-Leu-Pro-Leu-Pro-Arg (TWLPLPR) has been shown to ameliorate neuroinflammation and cognitive impairment by suppressing NLRP3 inflammasome activation and enhancing synaptic plasticity in both mice and HT-22 cells [14]. Some fermented food-derived products, such as lactobacillus plantarum C29-fermented soybean (DW2009), Lactobacillus pentosus var. plantarum-fermented defatted soybean, and Monascus-fermented red mold rice, were reported to alleviate AD progression and cognitive decline by regulating anti-inflammatory and antioxidant pathways [15,16,17]. Blockade of the NLRP3 inflammasome signaling reduces Aβ deposition and alleviates AD [18]. Our recent work identified Trp-Leu-Pro (WLP), a novel antioxidant peptide derived from the sea squirt (*Halocynthia roretzi*), which mitigated 6-OHDA-induced oxidative stress, suggesting its potential for alleviating neurodegenerative diseases [19]. However, whether WLP can mitigate AD pathology and its underlying mechanisms remains to be elucidated.

Establishing human-relevant models is essential for investigating the neuroprotective effects of WLP in AD. Despite their widespread use, cellular and animal models face limitations, including species-specific differences and the lack of a physiologically relevant microenvironment [20]. Cerebral organoids, self-organizing 3D structures derived from human induced pluripotent stem cells (hiPSCs), provide a platform that mimics the cellular diversity and spatial organization of the human brain [21]. These organoids offer a unique opportunity to investigate pathological changes in human neurological diseases, including AD. Notably, hiPSC-derived cerebral organoid models have successfully replicated key pathological hallmarks of AD, such as Aβ plaque deposition and NFTs formed by misfolded tau proteins [22]. Given that the method is time-consuming and the limited availability of patient-derived samples, there is a highlighted need for more efficient approaches to establish AD models using cerebral organoids.

OKA is a microalgal toxin produced by marine dinoflagellates and certain sponges [23]. Consumption of contaminated shellfish, such as oysters, mussels, and scallops, can lead to the bioaccumulation of OKA in humans. Experimental evidence demonstrates that OKA promotes hyperphosphorylation through PP2A inhibition, subsequently inducing Aβ amyloid deposition, apoptotic cell death, and inflammatory responses [18,24]. Therefore, OKA has been widely used in the construction of AD models. OKA presents a rapid and efficient strategy for modeling AD pathology in cerebral organoids. In this study, we treated cerebral organoids with OKA and validated the model by assessing the presence of Aβ plaques and NFTs, which are primary hallmarks of AD.

This study aimed to establish an AD model in cerebral organoids using OKA, evaluate the neuroprotective effects of the food-derived peptide WLP against AD-associated pathologies, and explore the underlying mechanisms of these effects. The hiPSCs were used to generate cerebral organoids, which were treated with OKA to mimic AD-like pathology. Subsequently, the protective effects of WLP on OKA-induced pathological phenotypes and cell viability were then assessed. Furthermore, RNA sequencing was performed to preliminarily uncover the molecular mechanisms mediating WLP’s neuroprotective effects.

## 2. Materials and Methods

### 2.1. Chemicals

The tripeptide Trp-Leu-Pro (WLP) was chemically synthesized by GeneScript (Nanjing, China). A 3 mM stock solution of WLP was prepared and stored at −20 °C. OKA was obtained from MedChemExpress (Monmouth Junction, NJ, USA) and dissolved in ethanol to 124.2 μM, which was also stored at −20 °C. Prior to employment, the stock solutions were individually diluted to the requisite concentrations using the appropriate medium.

### 2.2. The hiPSCs Culture

The human iPSC line (NCCSEDi001-A, https://hpscreg.eu/cell-line/NCCSEDi001-A, accessed on 29 November 2023) was generated by employing Yamanaka’s retroviral transduction method on normal male skin fibroblasts. For details, refer to our previous study [25]. Its authenticity was rigorously validated through karyotyping, pluripotency assays, and trilineage differentiation tests [26]. The hiPSCs were cultured on Matrigel-coated 6-well plates with the mTeSR™1 medium changed daily. When confluence reached 70–80%, they were detached utilizing ReLeSR™ for passaging onto fresh plates.

### 2.3. Generation of Cerebral Organoids

The STEMdiff™ Cerebral Organoid Kit (STEMCELL Technologies, Vancouver, BC, Canada), along with previously published methods, served to generate cerebral organoids via a modified protocol [26]. Initially, hiPSCs were dissociated using TrypLE (Invitrogen, Carlsbad, CA, USA) and resuspended. Embryoid bodies (EBs) were formed by seeding 5000–6000 cells per well in a 96-well plate (Corning, 7007, New York, NY, USA) with 100 μL of EB Formation Medium. The medium was refreshed every other day and the day of inoculation was designated as day 0. On day 5, the EBs were transferred to 6-well plates (Corning, 3471, New York, NY, USA) and cultured in Induction Medium to induce neuroectodermal differentiation. On day 7, EBs were embedded in Matrigel and cultured in Expansion Medium for 3 days. The medium was subsequently switched to Differentiation Medium containing neurobasal (Gibco, Grand Island, NY, USA), 1% *v*/*v* Glutamax (Gibco, Grand Island, NY, USA), 1% *v*/*v* NEAA (Gibco, Grand Island, NY, USA), 2% *v*/*v* B-27 without vitamin A (Gibco, Grand Island, NY, USA), 20 ng/mL epidermal growth factor (STEMCELL Technologies, Vancouver, BC, Canada), and 20 ng/mL fibroblast growth factor 2 (STEMCELL Technologies, Vancouver, BC, Canada) for an additional 3 days. On day 14, the Differentiation Medium was replaced with Maturation Medium containing 1% *v*/*v* Glutamax, 1% *v*/*v* NEAA, 2% *v*/*v* B-27 without vitamin A, 20 ng/mL BDNF (PeproTech, Cranbury, NJ, USA), and 20 ng/mL NT3 (PeproTech, Cranbury, NJ, USA). From day 40 onward, BDNF and NT3 were excluded from the maturation medium to facilitate terminal maturation. The organoids were transferred to a shaker operating at 65 rpm and maintained at 37 °C, with the medium refreshed every 3 days to ensure adequate nutrient supply and promote further maturation. To monitor morphological and size changes, organoids were photographed at defined time points during the culture process.

### 2.4. OKA Treatment for Inducing AD-Related Pathologies in Cerebral Organoids

OKA, a potent polyether fatty acid toxin, is a selective inhibitor of protein phosphatases PP1 and PP2A. OKA is commonly used as a tool to induce tau hyperphosphorylation by inhibiting phosphatase activity, which in turn leads to AD-like pathology [27,28]. Moreover, previous studies have demonstrated that 48 h OKA exposure effectively induces AD-like pathology in neuronal models, including Aβ amyloid deposition and tau hyperphosphorylation [29,30]. In this study, 48 h OKA exposure was utilized to establish AD cerebral organoid models. OKA doses of 12.5, 25, 50, and 100 nM were tested, based on previous studies suggesting 0–100 nM as the optimal concentration range for inducing tau phosphorylation. Organoids were categorized into a control group and four OKA-treated groups. Three organoids were seeded per well, with three replicate wells per group). On day 44, the organoids were treated with corresponding solutions for 48 h. After treatment, the organoids were harvested for imaging and subsequent analyses to assess AD-related pathologies.

### 2.5. Administration Scheme of WLP on Cerebral Organoids for Assessing Its Neuroprotective Potential

The organoids were randomly divided into four experimental groups: a control group, a 3 mM WLP-treated group, a 25 nM OKA-treated group, and a 3 mM WLP + 25 nM OKA group. The 3 mM WLP and 25 nM OKA solution were prepared in maturation medium. For the WLP and OKA+WLP groups, organoids were pretreated with 3 mM WLP on day 42 for 48 h, followed by incubation with or without 25 nM OKA. After the addition of maturation medium, the final concentration of WLP was adjusted to 1.5 mM. For the OKA and OKA+WLP groups, 25 nM OKA was added on day 44 and incubated for 48 h. The control group received maturation medium on the same schedule. Following the treatments, organoids were harvested for morphological analysis, imaging, and further examinations to assess the effects of WLP on AD-related pathologies and neurotoxicity induced by OKA.

### 2.6. Immunofluorescence

Cerebral organoids were washed with phosphate-buffered saline (PBS) and fixed in 4% paraformaldehyde for 1 h. Subsequently, the organoids were washed again in PBS and dehydrated overnight in 30% *w*/*v* sucrose. The samples were then immersed in a 1:1 mixture of 2% *w*/*v* sucrose and optimal cutting temperature (OCT) solution, followed by an additional 4 h immersion in pure OCT. The organoids were immobilized within frozen OCT and sectioned into 20 μm slices using a cryostat (Leica, Chicago, IL, USA). The sections were blocked with a mixture containing 10% goat serum (Abbkine, Santa Ana, CA, USA) and 0.1% Triton X-100 (Sigma-Aldrich, St. Louis, MO, USA) in PBS at room temperature for 1 h. After blocking, the sections were incubated overnight with primary antibodies, previous to a 1 h incubation with corresponding secondary antibodies. The nuclei were counterstained with 4′,6-diamidino-2-phenylindole (DAPI) solution for 10 min in the dark. After thorough washing, the sections were mounted with an autofluorescence quenching sealer. Finally, the stained sections were visualized using a Leica SP8 confocal microscope, and quantitative analysis was performed using ImageJ software (version 1.54i). Detailed information regarding antibodies and DAPI solution is provided in Table S1.

### 2.7. TUNEL Assay

Apoptotic cells were labeled using TUNEL method according to the manufacturer’s instructions for the In Situ Cell Death Detection Kit (Roche, Basel, Switzerland). Briefly, a working solution prepared by mixing the enzyme and label solutions at a 1:9 ratio was employed to incubate the sections for 30 min. The sections were then mounted onto glass slides. Both image acquisition and quantitative analysis were consistent with procedures of immunofluorescence.

### 2.8. RNA Sequencing

RNA sequencing was conducted for the control, OKA-treated, and OKA+WLP groups as previously described [26]. Total RNA was extracted with TRIzol reagent (Invitrogen, CA, USA). The concentration and integrity of RNA samples were evaluated using NanoDrop2000c spectrophotometer (ThermoFisher Scientific, Waltham, MA, USA). mRNA was then isolated from total RNA using a sample preparation kit (Illumina, San Diego, CA, USA) and employed as a template for complementary DNA (cDNA) synthesis. Subsequently, the cDNA library was constructed and sequenced on an Illumina HiSeq 4000 platform. Based on the *p*-adjust value < 0.05 and |Fold change| ≥ 1.2, differentially expressed genes (DEGs) were identified. Functional enrichment analyses including Gene Ontology (GO) and Kyoto Encyclopedia of Genes and Genomes (KEGG) pathway analyses were subsequently performed on the identified DEGs. The RNA-sequencing assay was conducted by Majorbio Biopharm Technology Co, Ltd. (Shanghai, China).

### 2.9. Quantitative Real-Time PCR (qPCR)

qPCR was conducted to evaluate the expression levels of *HMOX1* and *EPAS1*. To extract an adequate quantity of RNA, a consistent sample size of 3 to 4 organoids was used per group. In accordance with our published study, total RNA was extracted employing the miRNeasy Mini Kit (QIAGEN, Redwood City, CA, USA). Thereafter, cDNA was synthesized via reverse transcription utilizing the TransScript First-Strand cDNA Synthesis SuperMix (Transgen, Shanghai, China). The primer sequences were as follows: HMOX1-F (ATGCCCCAGGATTTGTCAGA), HMOX1-R (AAGTAGACAGGGGCGAAGAC), EPAS1-F (TCGGAGAGGAGGAAGGAGAA), and EPAS1-R (GAGGAGAGGAGCTTGTGTGT). The gene accession identifiers are as follows: *EPAS1* (AC016696) and *HMOX1* (AY460337). Subsequently, qPCR was performed using Bestar qPCR SYBR Green MasterMix (DBI Bioscience, Shanghai, China). GAPDH served as the reference gene for normalization, and the relative expression of the target genes was calculated using the 2^−ΔΔCt^ method.

### 2.10. Statistical Analysis

All experiments were conducted in triplicates. Quantitative measurements of immunofluorescence images were conducted using ImageJ software. Statistical analyses and data visualization were carried out using GraphPad Prism (version 9). Data are expressed as mean ± standard deviation (mean ± SD). One-way ANOVA followed by Tukey’s post hoc analysis was employed to analyze the data and statistical significance was evaluated based on a *p*-value < 0.05.

## 3. Results

### 3.1. Generation and Identification of hiPSC-Derived Cerebral Organoids

**Figure 1:**
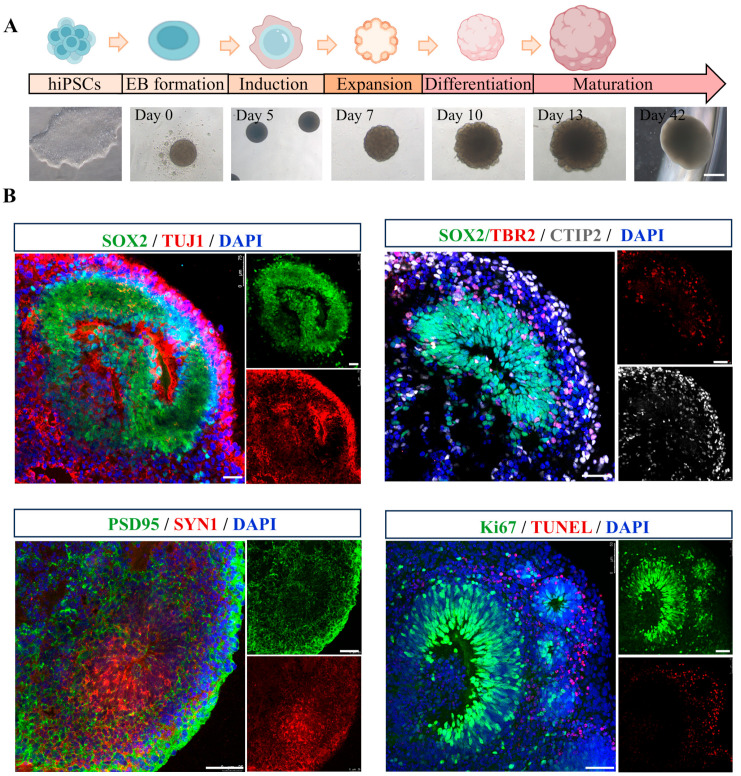
Generation and characterization of hiPSC-derived cerebral organoids. (**A**) A schematic representation of the protocol for the generation of hiPSC-derived cerebral organoids, accompanied by representative phase-contrast images illustrating various developmental stages. Scale bar, 1 mm. (**B**) Representative immunofluorescence images showing the expression of neural progenitor marker SOX2 (green), neuronal marker TUJ1 (red), intermediate progenitors maker TBR2 (red), cortical deep layer marker CTIP2 (gray), presynaptic marker SYN1 (red), postsynaptic marker PSD95 (green), proliferation marker Ki67 (green), and apoptosis maker TUNEL (red) in cerebral organoids. Scale bar, 50 μm.

Cerebral organoids were generated following a previously established protocol [31], of which morphological alterations were photographed at multiple time points (day 0, 5, 7, 10, 13, and 42) (Figure 1A). By day 40, the organoids exhibited multiple cortical rosette-like structures, indicative of advanced neural differentiation. To assess the feasibility of this 3D model, cerebral organoids were analyzed for the expression of various specific markers. As shown in Figure 1B, SOX2^+^ neural progenitor cells (NPCs) were observed in ventricular zone-like (VZ) layer, while TUJ1^+^ neurons were detected in neuronal layer, demonstrating the cellular diversity within cerebral organoids. Immunohistochemical staining further validated the expression of the cortical layer marker CTIP2. Additionally, the presence of mature neurons was confirmed through immunostaining for pre- and post-synaptic markers, SYN1 and PSD95. Proliferation and apoptosis rates were assessed using Ki67 and TUNEL staining, respectively. The results revealed an increased population of proliferating cells and a reduction in the incidence of apoptotic cells within the cerebral organoids, indicative of their favorable growth and viability. Collectively, these findings suggest that the cerebral organoids generated in this study are derived from hiPSCs, exhibit well-developed VZ-like regions, and encompass a diverse array of neuronal cell types, making them highly suitable for subsequent experimental investigations.

### 3.2. Establishment of an OKA-Induced AD Model in Cerebral Organoid

**Figure 2:**
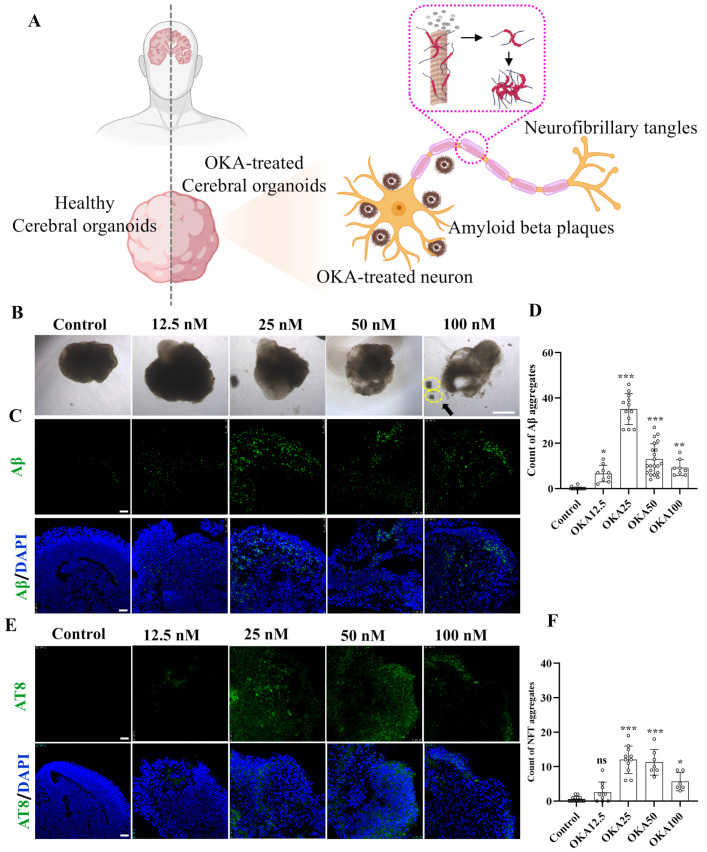
Establishment of OKA-induced AD pathologies in cerebral organoid models. (**A**) Schematic representation of modeling AD-like pathologies in cerebral organoids using OKA, highlighting the development of Aβ plaques and NFTs in neurons. (**B**–**D**) Bright-field images of cerebral organoids and representative immunostaining of Aβ deposition following 48 h of OKA exposure from 12.5 nM to 100 nM. Scale bar, 1 mm (bright-field images) and 25 μm (immunostaining). The yellow dashed circles highlight the fragmentation of the organoid structures. Quantitative analysis of Aβ aggregates is displayed in panel D. (**E**,**F**) Representative immunostaining of NFTs (AT8) in cerebral organoids after 48 h of OKA treatment at varying concentrations (12.5–100 nM). Scale bar, 25 μm. Quantification of NFT aggregates is shown in panel F. Data are expressed as mean ± standard deviation (*n* = 6). Statistical significance was assessed using one-way ANOVA, followed by post hoc analysis: * *p* < 0.05, ** *p* < 0.01, and *** *p* < 0.001; ns, not significant.

The schematic diagram in Figure 2A illustrates the experimental design for inducing AD-like pathology in cerebral organoids using OKA, highlighting the formation of two key neuropathological hallmarks: insoluble extracellular Aβ plaques and intracellular NFTs. Forty-two-day-old cerebral organoids were exposed to varying concentrations of OKA (ranging from 0 to 100 nM) for a duration of 48 h. Then, the organoids were harvested and analyzed using immunofluorescence staining.

The cerebral organoids from the control group retained their structural integrity and normal size. In contrast, those treated with OKA exhibited a significant reduction in size, compromised structural integrity, and irregular, frizzy edges (Figure 2B). A high dose of OKA (50–100 nM) resulted in a substantial presence of cellular debris, indicating extensive cell death. As shown in Figure 2C, the control group exhibited no detectable Aβ accumulation. Conversely, organoids treated with OKA (12.5–100 nM) displayed significantly elevated Aβ levels, with the highest accumulation observed in the 25 nM group. However, Aβ accumulation levels decreased dose-dependently in the 50–100 nM OKA-treated groups, likely due to excessive OKA concentrations causing organoid disintegration and widespread neuronal loss. These findings suggest that OKA concentrations of 50–100 nM are unsuitable for subsequent modeling.

Tau hyperphosphorylation, a critical precursor to NFT formation, facilitates the aggregation of tau into insoluble paired helical filaments (PHFs), which subsequently form NFTs [32]. To assess tau phosphorylation, organoids treated with OKA at various concentrations were immunostained with AT8, a monoclonal antibody specific to PHF-tau and abnormally phosphorylated tau. As depicted in Figure 2E, the pattern of tau hyperphosphorylation across the dosage groups mirrored the Aβ accumulation results, with the 25 nM OKA group showing the highest NFT formation. Increasing OKA concentrations beyond 25 nM led to a dose-dependent reduction in NFT formation (Figure 2F), consistent with findings by Derya Metin-Armağan and Ludovic Martin, who demonstrated that 25 nM OKA induces robust tau hyperphosphorylation in neuronal cells [33,34]. These results confirm that 25 nM OKA is an optimal concentration for inducing AD-like pathologies in cerebral organoids.

To sum up, we exposed 42-day-old cerebral organoids to 0, 25, 50, and 100 nM OKA for 48 h. Immunostaining and bright-field images indicated that 25 nM OKA is an optimal concentration for inducing AD-like pathologies in cerebral organoids.

### 3.3. Effects of WLP on Cell Viability and Neuronal Differentiation in OKA-Induced Cerebral Organoids

Our previous study reported that the antioxidant peptide WLP at concentrations ranging from 0.75–3.00 mM did not exhibit cytotoxic effects on PC12 cells. Moreover, preincubation with WLP demonstrated significant cytoprotective effects against 6-OHDA-induced oxidative stress in PC12 cells [19]. Considering the differences between models and treatments, OKA-induced cerebral organoids were exposed to 0.75, 1.5, and 3 mM WLP for 48 h in preliminary experiments. As illustrated in Figure S1, no significant differences were observed between 1.5 mM and 3.0 mM WLP in terms of Aβ and NFT aggregation in AD cerebral organoids. Therefore, 1.5 mM WLP was selected for subsequent experiments.

**Figure 3:**
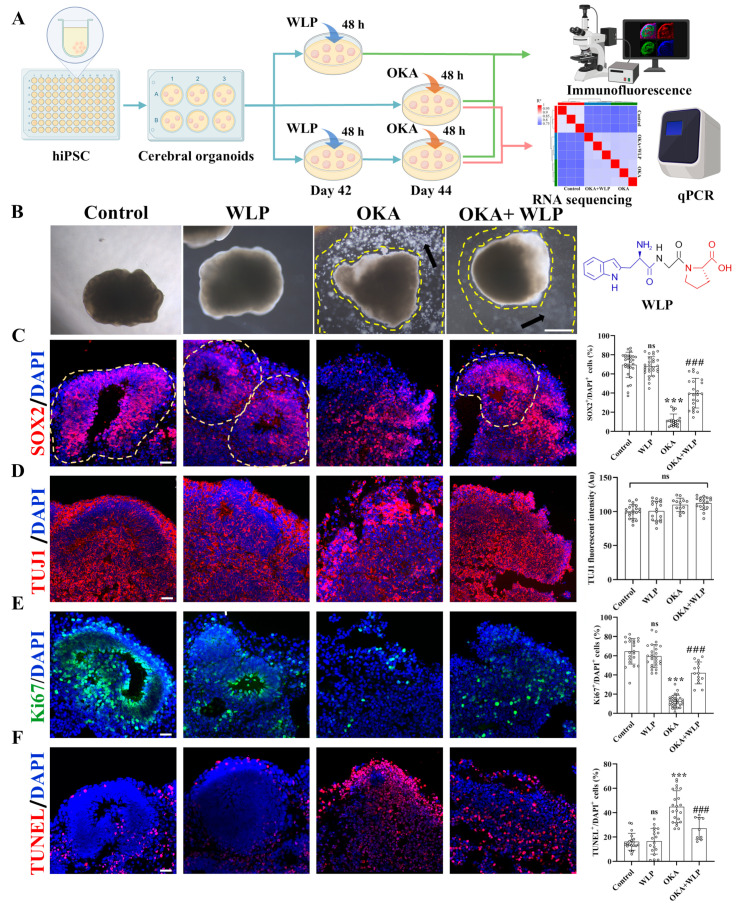
Effects of WLP on neuronal differentiation and cell viability of OKA-induced cerebral organoids. (**A**) Schematic representation of the experimental workflow for chemical exposure to OKA and the food-derived peptide WLP. (**B**) Bright-field images of cerebral organoids following OKA treatment. Scale bars, 1 mm. Arrows indicate regions between the dashed lines, highlighting areas of cellular fragments. (**C**–**F**) Representative immunostaining images for SOX2, TUJI, Ki67, and TUNEL in control, WLP-treated, OKA-treated, and OKA+WLP-treated groups. Quantification of SOX2^+^, Ki67^+^, and TUNEL^+^ cells as a percentage of DAPI^+^ cells, along with TUJ1 fluorescent intensity in VZ-like regions of cerebral organoids (*n* = 6 VZ-like areas/group). Inferred VZ is delineated by dashed lines. Statistical significance was assessed using one-way ANOVA, followed by post hoc analysis. The scale bar represents 25 μm. * indicates the statistical significance relative to the control group, while # denotes the statistical significance relative to the OKA-treated group *** *p* < 0.001; ### *p* < 0.001; ns, not significant.

To explore the neuroprotective effects of WLP, we divided the organoids into the following four groups: the control, WLP-treated, OKA-treated, and OKA+WLP groups (Figure 3A). Organoids from the control and WLP-treated groups retained their structural integrity and exhibited multiple VZ-like regions under a bright-field microscope (Figure 3B). In contrast, organoids exposed to 25 nM OKA appeared smaller with frayed edges and a significant presence of cellular debris, indicating neural damage. Pretreatment with WLP notably reduced cellular debris in the OKA+WLP group, suggesting a protective effect. To evaluate this further, cerebral organoids were collected on day 44 and stained for neural-specific markers. SOX2, indicative of neuronal progenitor cells (NPCs), and TUJ1, a marker for neurons, were assessed. The OKA treatment significantly reduced the percentage of SOX2^+^ cells (11.65 ± 6.48%) compared to the control (69.54 ± 13.14%) and disrupted VZ-like areas (Figure 3C), confirming its neurotoxic effects. In contrast, the OKA+WLP group exhibited a significant recovery in SOX2^+^ cells (39.90 ± 15.49%). In addition, the average fluorescence intensity of TUJ1 in VZ-like regions was similar across the control, OKA-treated, and WLP-treated groups (Figure 3D), indicating that while OKA did not substantially alter neuron numbers, it compromised their structural integrity.

To further assess OKA and WLP’s effects, cell proliferation and apoptosis were analyzed within VZ regions. Immunofluorescence revealed a significant reduction in Ki67^+^ cells in the OKA-treated group compared to the control (Figure 3E), while no differences were noted between the control and WLP-treated groups. TUNEL staining demonstrated a higher incidence of apoptotic cells in the VZ regions of OKA-treated organoids, whereas no significant differences were detected between the control and WLP-treated groups (Figure 3F). These findings confirm that OKA suppresses neural cell proliferation and induces apoptosis in cerebral organoids. Furthermore, the 1.5 mM WLP was non-toxic to cerebral organoids. Compared to the OKA-treated group, the OKA+WLP-treated group showed a marked increase in Ki67^+^ cells (from 12.53 ± 6.35% to 42.13 ± 11.39%) and a significantly reduction in apoptotic cells (from 44.89 ± 13.14% to 27.11 ± 9.02%).

Collectively, immunostaining showed that the OKA+WLP-treatment partially restored SOX^+^ cell populations, increased Ki67^+^ cells, and reduced TUNEL^+^ cells. The results suggested that WLP mitigates OKA-induced apoptosis and provides neuroprotection to cerebral organoids.

### 3.4. WLP Ameliorated OKA-Induced Aβ-like Pathology and p-Tau Elevation in Cerebral Organoids

**Figure 4:**
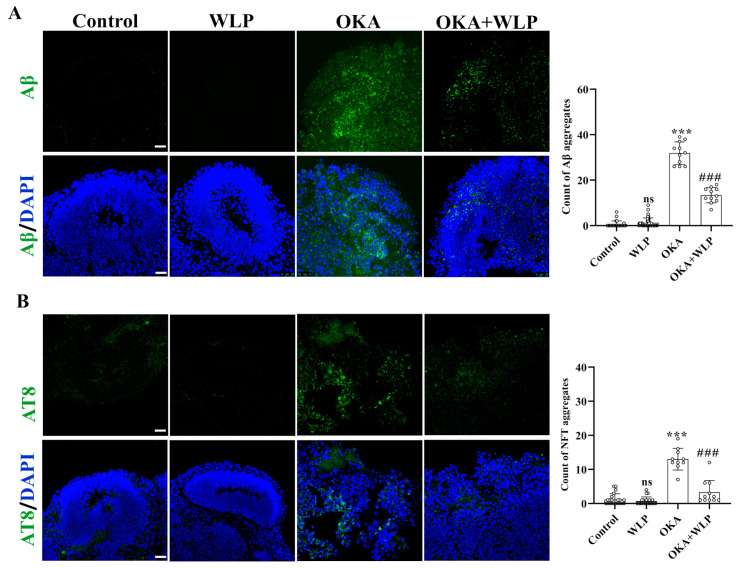
Protective effect of WLP peptide on OKA-induced AD pathologies in cerebral organoids. (**A**,**B**) Representative immunostaining images of Aβ and AT8 (a marker of phosphorylated tau) in control, WLP-treated, OKA-treated, and OKA+WLP-treated cerebral organoids. Scale bars, 25 μm. Results are presented as mean ± SD (*n* = 6). Statistical significance was assessed using one-way ANOVA, followed by post hoc analysis, with *p*-values denoted as *** *p* < 0.001; ### *p* < 0.001; ns, not significant. *** represents the statistical significance compared to the control group, while ### denotes the statistical significance compared to the OKA-treated group.

To evaluate the protective effects of WLP on OKA-induced Aβ-like pathology, immunostaining for Aβ and AT8 was performed. No significant differences were observed in Aβ and NFT aggregation between the control and WLP-treated groups (Figure 4A,B). In contrast, OKA-treated cerebral organoids showed a marked increase in Aβ and NFT aggregate, with levels rising to 31.91 ± 4.93% and 13.00 ± 3.20%, respectively, compared to the control. Notably, pretreatment with WLP significantly reduced Aβ and NFT accumulation in the OKA+WLP group, with levels decreasing to 13.36 ± 3.36% and 3.27 ± 3.47%, respectively (*p* < 0.001). These findings suggest that WLP effectively mitigates p-tau hyperphosphorylation and NFT formation in OKA-induced cerebral organoids. This highlights the potential neuroprotective effects of the food-derived peptide WLP in alleviating AD-associated pathologies.

### 3.5. DEGs Analysis of OKA-Induced Cerebral Organoids Pretreated with the WLP

**Figure 5:**
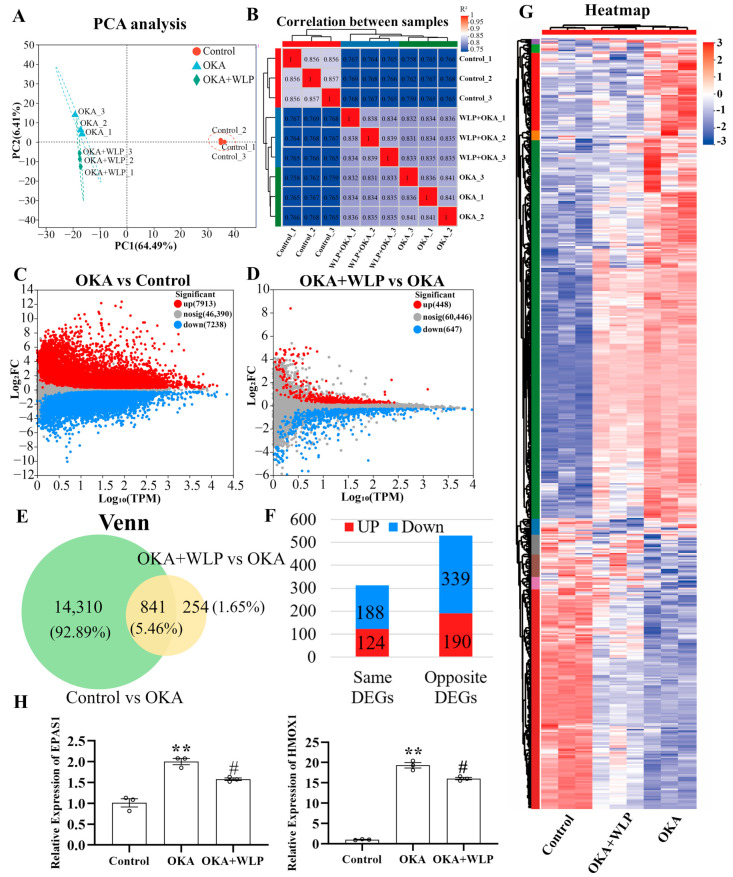
Analysis of DEGs in OKA-treated cerebral organoids with or without the food-derived peptide WLP. (**A**) PCA illustrating the clustering of cerebral organoid samples treated with OKA and/or WLP, highlighting group-specific variability. (**B**) Correlation analysis reveals greater similarity among samples within the same treatment groups compared to samples from different groups. (**C**) A volcano plot illustrating the DEGs between the control and OKA-treated groups. (**D**) Volcano plot showing DEGs between the OKA-treated and OKA+WLP-treated groups. (**E**,**F**) Venn diagram illustrating 841 shared DEGs among the control, OKA-treated, and OKA+WLP-treated groups. (**G**) Heat map visualizing the expression profiles of 841 shared DEGs across the control, OKA-treated, and OKA+WLP-treated groups. (**H**) Validation of gene expression using qRT-PCR. ** indicates statistical significance compared to the control group, while # indicates the statistical significance compared to the OKA-treated group. Statistical significance was assessed using one-way ANOVA, followed by post hoc analysis: ** *p* < 0.01, # *p* < 0.05.

To explore the mechanisms underlying the neuroprotective effects of WLP, RNA sequencing was performed. After filtering low-quality reads, a total of 61.69 Gb of clean reads were obtained for further analysis. Among these, 95.46–96.75% aligned with the Homo sapiens genome. Principal component analysis (PCA) clearly showed variability in the nine samples from the control, OKA-treated, and OKA+WLP-treated groups (Figure 5A), with the first two components accounting for 6.41% and 64.49% of the total variance, respectively. Correlation analysis further demonstrated distinct differences among the three groups (Figure 5B).

The volcano plot identified 15,151 DEGs in the OKA-treated group relative to the control, with 7913 genes up-regulated and 7238 genes down-regulated (Figure 5C). Similarly, 1135 DEGs were detected in the OKA+WLP group compared to the OKA-treated group, comprising 488 up-regulated and 647 down-regulated genes (Figure 5D). From those, 841 genes exhibiting opposing expression treads between the OKA+WLP- and OKA-treated groups were selected to construct a gene set (Figure 5E). Specifically, 190 genes were up-regulated in the OKA+WLP group but down-regulated in the OKA-treated group, while 339 genes displayed reverse trend (Figure 5F). These 529 DEGs with contrasting expression patterns were subjected to further analysis (Table S2). Hierarchical clustering indicated different responses of cerebral organoids to the OKA and OKA+WLP treatments (Figure 5G).

To validate the RNA sequencing results, two oxidative stress-associated genes, *HMOX1* and *EPAS1*, were selected for qPCR analysis. As shown in Figure 5H, the mRNA level of *HMOX1* and *EPAS1* were significantly elevated following OKA treatment compared to the control but were markedly down-regulated in the OKA+WLP group, further supporting the neuroprotective role of WLP.

RNA sequencing was conducted in the control, OKA-treated, and OKA+WLP groups. Briefly, 529 DEGs with completely opposite expression patterns in the OKA-treated and OKA+WLP groups were identified. Additionally, qPCR analysis validated the RNA sequencing results.

### 3.6. GO and KEGG Pathway Enrichment Analysis of Up- and Down-Regulated DEGs

**Figure 6:**
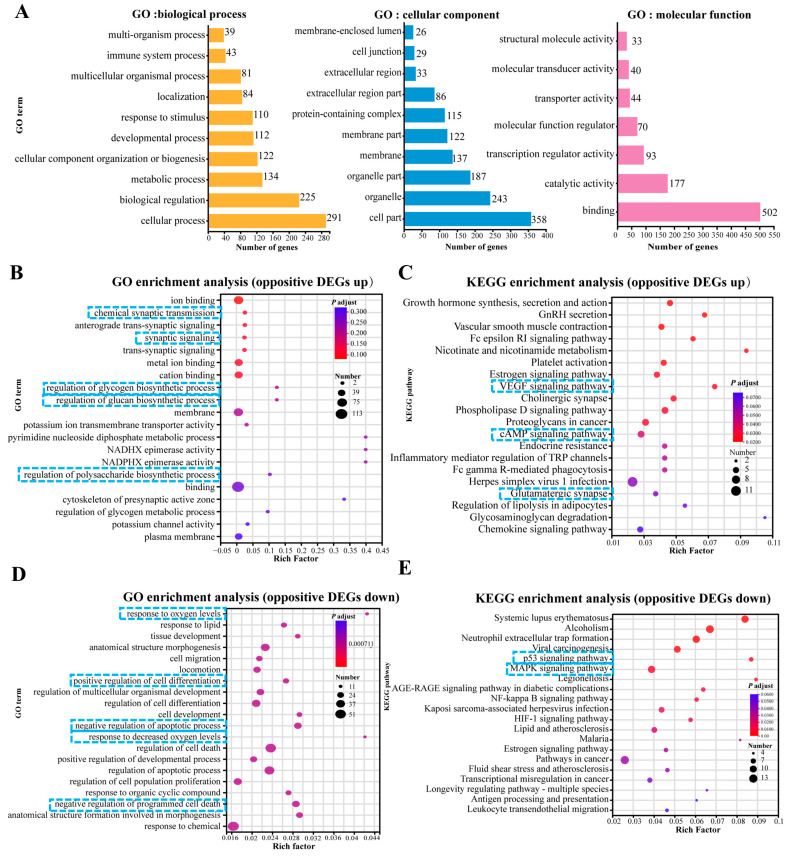
Top 20 GO and KEGG pathway enrichment analyses of up-regulated and down-regulated DEGs. (**A**) GO enrichment analysis of DEGs categorized under level 1 and level 2 GO terms. (**B**,**C**) GO enrichment analysis (**B**) and KEGG pathway enrichment analysis (**C**) of 190 up-regulated DEGs. (**D**,**E**) GO enrichment analysis (**D**) and KEGG pathway enrichment analysis (**E**) of 339 down-regulated DEGs. In panels (**B**–**E**), the enrichment factor reflects the ratio of DEGs to the total number of genes identified within each pathway. Color intensity corresponds to the adjusted *p*-value, with higher values in blue and lower values in red. Lower adjusted *p*-values indicate higher enrichment significance. The size of the dots represents the number of DEGs associated with each pathway. The blue squares highlight the key processes and pathways.

To elucidate the biological response to OKA and WLP exposure in cerebral organoids, we performed enrichment analysis focusing on biological processes, cellular components, and molecular functions (Figure 6A). After the OKA and WLP treatments, the DEGs were predominantly associated with ten GO terms (level 2) within the biological process category, including response to stimulus, development process, cellular component organization or biogenesis, metabolic process, biological regulation, and cellular process. Within the cellular component category, the DEGs were primarily enriched in four GO terms (level 2): cell part, organelle, organelle part, and protein-containing complex. In terms of molecular function, binding and catalytic activity emerged as the top two GO terms (level 2).

Furthermore, the GO analysis showed that 190 up-regulated DEGs were mainly enriched in “chemical synaptic transmission (GO:0007268, *p* = 2.78 × 10^−5^)”, “synaptic signaling (GO:0099536, *p* = 3.99 × 10^−5^)”, “metal ion binding (GO:0046872, *p* = 3.37 × 10^−5^)”, “regulation of glycogen biosynthetic process (GO:0005979, *p* = 1.08 × 10^−4^)”, “regulation of glucan biosynthetic process (GO:0010962, *p* = 1.08 × 10^−4^)”, and “regulation of polysaccharide metabolic process (GO:0032881, *p* = 3.7 × 10^−4^)” (Figure 6B). KEGG pathway enrichment analysis for these 190 DEGs highlighted the top 20 significantly enriched pathways, as shown in Figure 6C. Prominent pathways included the VEGF signaling pathway, cholinergic synapse, cAMP signaling pathway, and glutamatergic synapse.

For the 339 DEGs that were down-regulated in the OKA-treated group but up-regulated in the OKA+WLP group, GO analysis revealed significant associations with pathways such as “response to oxygen levels (GO:0070482, *p* = 2.41 × 10^−7^)”, “negative of cell apoptotic process (GO:0043066, *p* = 8.23 × 10^−7^)”, “response to decreased oxygen level (GO:0036293, *p* = 8.44 × 10^−7^)”, “positive regulation of developmental process (GO:0051094, *p* = 9.02 × 10^−7^)”, “positive regulation of cell differentiation (GO:0045597, *p* = 6.09 × 10^−7^)”, and “negative regulation of programmed cell death (GO:0043069, *p* = 6.09 × 10^−6^)” (Figure 6D). KEGG pathway enrichment analysis for these 339 DEGs identified the top 20 significantly enriched pathways (Figure 6E), which were strongly correlated with longevity regulation and cellular senescence, including the estrogen signaling pathway, P53 signaling pathway, and MAPK signaling pathway.

**Figure 7:**
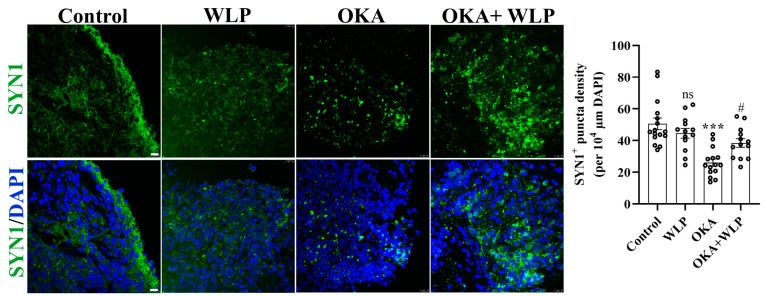
Representative immunostaining images of SYN1 in control, OKA-treated, and OKA+WLP-treated cerebral organoids. Scale bars, 25 μm. Statistical significance was assessed using one-way ANOVA, followed by post hoc analysis. *** represents the statistical significance compared to the control group, while # denotes the statistical significance compared to the OKA-treated group. *** *p* < 0.001; # *p* < 0.05; ns, not significant.

To validate the transcriptomic analysis related to the synaptic pathway, we employed immunostaining of SYN1^+^ presynaptic puncta (Figure 7). Consistent with the transcriptomic findings, we discovered that pre-treatment with WLP could partially reverse the reduction in SYN1^+^ presynaptic puncta density induced by OKA. Collectively, the results confirmed the potential regulatory role of WLP in synaptic function under OKA exposure, which is crucial for understanding the underlying mechanisms of WLP’s neuroprotective effects.

In conclusion, RNA sequencing analysis showed that the protective effect of food-derived WLP was related to the cholinergic synaptic signaling, glutamatergic synapse, and oxidative stress attenuation pathways.

## 4. Discussion

AD, the most prevalent neurodegenerative disorder worldwide, represents a critical public health burden. Current pharmacological therapies, including donepezil, memantine, galantamine, and rivastigmine, can alleviate the progression of AD, yet they are associated with adverse effects such as dizziness and nausea [35]. Previous studies have demonstrated that food-derived peptides are highly safe and possess the potential to prevent AD [6]. In our study, the food-derived tripeptide WLP exhibits superior safety, and its short peptide chain and low molecular weight suggest that it may penetrate the blood-brain barrier more efficiently than long-chain peptides [36]. Therefore, the food-derived WLP in our study is a promising candidate for the prevention of AD.

In this study, we established an AD model using hiPSC-derived cerebral organoids treated with 25 nM OKA. The OKA-exposed organoids successfully recapitulated key pathological hallmarks of AD, including Aβ-like pathology, elevated levels of NFTs, and neuronal loss. Previous studies have demonstrated the potential of cerebral organoids to model AD-like pathological features. For instance, hiPSC-derived cerebral organoids were treated with serum from AD patients for 12 days, resulting in increased levels of insoluble Aβ and NFTs, alongside synaptic loss and reduced neural network activity [37]. Nevertheless, serum’s complexity and heterogeneity, containing various bioactive components, may influence cellular behavior and contribute to individual variability [37]. Pavoni et al. utilized small molecule inducers, such as the Aβ inducer Aftin-5, to elicit AD-like pathologies in organoids, exhibiting a significant increase in Aβ accumulation and neuronal loss, but failed to induce substantial changes in p-tau levels [38]. Another approach involved reprogramming fibroblasts from AD patients into hiPSCs to generate cerebral organoids. These organoids exhibited increased cellular apoptosis, reduced synaptic protein levels, and high levels of Aβ and p-tau proteins compared to those derived from healthy individuals [39]. However, this method is time-intensive, requiring 4–6 months for the organoid culture, and is associated with significant costs [40,41]. OKA has been extensively used in animal and cellular models to replicate AD neuropathology due to its ability to induce tau hyperphosphorylation and Aβ aggregation [42,43,44]. Despite its widespread use, limited studies have employed OKA in cerebral organoids. In this study, we pioneered the rapid construction of AD-like cerebral organoids using OKA, achieving model development within 48 h. This approach not only significantly reduces time and cost but also ensures the reproducibility and consistency of the model quality. Notably, we found that the AD organoid model constructed in our study exhibits significant similarities to previous research in cellular distribution and gene expression. Similar to AD iPSC-derived cerebral organoids, our AD cerebral organoids showed a comparable mean fluorescence intensity of TUJ1 to the control group and exhibited a more disordered distribution [39]. Furthermore, our AD organoid model showed decreased puncta density of the SYN1^+^ presynapse, which is consistent with AD patient serum-exposed organoids [37]. Besides the similarities in cellular distribution, our OKA-treated AD organoid model demonstrated gene expression consistent with previous studies, as transcriptomic analysis revealed the down-regulation of synaptic-related genes and up-regulation of oxygen-associated genes [37]. Collectively, the similarities in AD-like phenotypes, cellular distribution, and gene expression, support the validity and reliability of our established model, demonstrating its utility in disease mechanism studies and therapeutic development.

Food-derived peptides are characterized by their broad availability and minimal side effects, making them a promising avenue for neuroprotective strategies [7,11]. In our previous studies, we identified a novel peptide, WLP, derived from protein-rich sea squirts (*Halocynthia roretzi*) through protamex enzymolysis. This peptide exhibited notable antioxidant properties and demonstrated protective effects against 6-OHDA-induced neurotoxicity [19]. The antioxidant efficacy of WLP is hypothesized to stem from its tryptophan (Trp) content, as Trp is recognized as one of the most effective free amino acid for neutralizing AAPH-induced peroxyl radicals [45]. In this study, we explored the potential neuroprotective effects of WLP in an OKA-induced AD cerebral organoid model and sought to elucidate the underlying mechanisms. The findings revealed that pretreatment with the WLP significantly reduced the proportion of apoptotic cells while enhancing the proportion of proliferative cells within the AD cerebral organoid model. Furthermore, WLP markedly increased the proportion of neuronal progenitor cells, countering the detrimental effects of OKA exposure. Additionally, WLP mitigated the hyperphosphorylation of the tau protein and inhibited NFT formation in OKA-induced cerebral organoids, underscoring its potential as a therapeutic agent for AD-related pathologies.

Interestingly, we noted that the proportion of SOX2 significantly decreased after OKA treatment. This depletion could be attributed to an increase in the death of NPCs or the depletion of NPCs resulting from accelerated differentiation [46]. Subsequent quantification of TUJ1 fluorescence intensity revealed comparable neuronal βIII-tubulin expression levels between the control and OKA-treated groups. Nevertheless, this observation could be confounded by enhanced NPC differentiation potentially offsetting OKA-induced neuronal loss. Moreover, compared to controls, OKA elicited a remarkable increase in the proportion of TUNEL^+^ apoptotic cells. Notably, the increase was accompanied by a disruption of spatial distribution, suggesting impairment of the VZ-like regions. Consistent with previous studies [47,48], our findings suggest that OKA may induce apoptosis in multiple neural cell types, potentially resulting in the observed disrupted distribution and structural disorganization.

In the OKA+WLP-treated group, 190 genes were found to be up-regulated compared to the OKA-treated group, where these genes were down-regulated. GO-based functional enrichment analysis indicated that WLP exerts its neuroprotective effects primarily by modulating apoptosis and promoting cellular differentiation. Additionally, KEGG enrichment analysis of those 190 DEGs revealed that WLP’s neuroprotective action is mainly mediated through the up-regulation of synaptic-related pathways. Synapses, as vital units for signal processing, are crucial for higher cognitive functions, and synaptic defects are closely linked to neurodegenerative diseases like AD [49]. Among the various pathological changes observed in the brain of AD patients, synaptic loss exhibits the strongest correlation with cognitive decline [50]. Evidence suggests that the accumulation of Aβ and p-tau at synaptic sites exerts direct toxic effects on synapses, contributing to cognitive impairments in animal models [51,52,53]. Studies have shown that applying anti-Aβ antibodies to AD mouse models restores presynaptic marker density and reverses synaptic loss [54,55]. Given the critical role of synaptic weakening and loss in AD progression, strategies aimed at enhancing synaptic function and transmission strength are promising therapeutic approaches.

The expression of 339 genes was up-regulated in the OKA-treated group but down-regulated in the OKA+WLP-treated group. GO enrichment analysis revealed that these differentially down-regulated genes were primarily associated with oxidative stress and apoptosis-related pathways. Oxidative stress represents a critical imbalance between the production of reactive oxygen species (ROS), reactive nitrogen species (RNS), and antioxidant defenses and it is widely recognized as a significant contributor to the onset and progression of AD [56]. Excessive ROS causes oxidative damage to DNA, which facilitates the formation of NFTs that are characteristic of neurodegenerative diseases [57]. WLP may mitigate typical AD symptoms by modulating the REDOX pathway, thereby exerting protective effects in the AD cerebral organoid model. Additionally, KEGG enrichment analysis indicated that the food-derived peptide WLP could inhibit apoptosis, attenuate cellular senescence, and promote cell longevity via the MAPK and p53 signaling pathways, ultimately ameliorating pathological features of AD. The MAPK family, comprising terminal kinases such as c-Jun N-terminal kinase (JNK), p38 MAPK, and extracellular signal-regulated kinases (ERK), plays a crucial role in regulating diverse cellular processes, including proliferation, differentiation, and apoptosis [58]. The MAPK pathway is activated by ROS, such as hydroxyl radicals, superoxide anions, and hydrogen peroxide, which subsequently modulate tau protein phosphorylation levels by elevating β-secretase and γ-secretase activities, leading to increased Aβ deposition and NFT formation [59,60]. Furthermore, while the p53 protein plays a pivotal role in regulating mitochondria-mediated cell death, its hyperactivity has been implicated in several neurodegenerative disorders, including AD.

However, there are still limitations in our study. Despite our confirmation of the neuroprotective effect of WLP against AD, it is important to emphasize that this protective effect is restricted. On the one hand, while WLP pretreatment partially reversed OKA-induced reduction in the number of SOX2^+^ neural progenitor cells and Ki67^+^ proliferating cells, the distribution of SOX2^+^ and Ki67^+^ in the OKA+WLP group remained scattered. On the other hand, the OKA+WLP group did not display an intact VZ region as the control group did, collectively indicating WLP’s limited neuroprotection. Further, due to the lack of data on the absorption and distribution of WLP in both systemic circulation and the brain, its neuroprotective effects should be cautiously interpreted. Moreover, while the 1.5 mM WLP concentration used in this study demonstrated neuroprotective efficacy, systemic administration will require nanocarrier-mediated delivery. Accordingly, future studies will prioritize structural modifications to WLP, including lipid conjugation and disulfide bond incorporation, to enhance blood–brain barrier permeability and metabolic stability, thereby achieving therapeutic concentrations in vivo.

In addition, the cerebral organoid model in our study still requires further improvement. Firstly, organoid differentiation protocols should be standardized to minimize the intrinsic variation across organoid batches, which is a commonly acknowledged challenge in the organoid field [61]. Secondly, the current cerebral organoid model lacks microglia, whose activation has been demonstrated to accelerate the progression of AD via excessive neuroinflammation [62]. Finally, the organoid model we employed remains at a relatively early stage of maturation, with a limited variety of neural cell types and suboptimal neural function, particularly in complex electrophysiology. In future research, we intend to employ microglia-incorporated and more mature cerebral organoids. Moreover, we will utilize multielectrode array (MEA) to detect electrophysiological alterations to further explore the neuroprotective functions of WLP.

## 5. Conclusions

In conclusion, this study introduces a rapid and efficient approach for constructing AD cerebral organoids with consistent quality using OKA exposure. The resulting organoids successfully recapitulated key pathological features of AD, including Aβ-like plaques, elevated NFTs, and neuronal loss. The food-derived peptide WLP demonstrated significant neuroprotective effects by reducing Aβ and NFT levels in the AD cerebral organoid model. Transcriptomic analysis elucidated the underlying molecular mechanisms of WLP’s protective effects, emphasizing the modulation of synapse-related pathways, oxidative stress, and apoptosis. These findings suggest that WLP holds promise as a potential food-derived therapeutic candidate for AD prevention.

## Data Availability

The authors confirm that the data supporting the findings of this study are available within the article and its Supplementary Materials.

## Supplementary Materials

The following supporting information can be downloaded at: https://www.mdpi.com/article/10.3390/antiox14050553/s1, Figure S1. Immunostaining for Aβ and NFTs in OKA- and WLP-treated groups; Table S1. The list of antibodies used in this study; Table S2. Differential gene expression among the control, OKA, and WLP groups (gene set).

## Abbreviations

The following abbreviations are used in this manuscript:

AD	Alzheimer’s disease
OKA	okadaic acid
hiPSC	pluripotent stem cell
Aβ	amyloid-beta
NFT	neurofibrillary tangles
