# Supplementary material for: Sea Squirt-Derived Peptide WLP Mitigates OKA-Induced Alzheimer’s Disease-like Phenotypes in Human Cerebral Organoid
Source: Antioxidants (Basel). 2025 May 7;14(5):553. doi: 10.3390/antiox14050553 (PMC12108538; doi:10.3390/antiox14050553)
Supplement: Supplementary file 1 [file antioxidants-14-00553-s001.zip › antioxidants-3537044-supplementary.pdf]

Figure S1

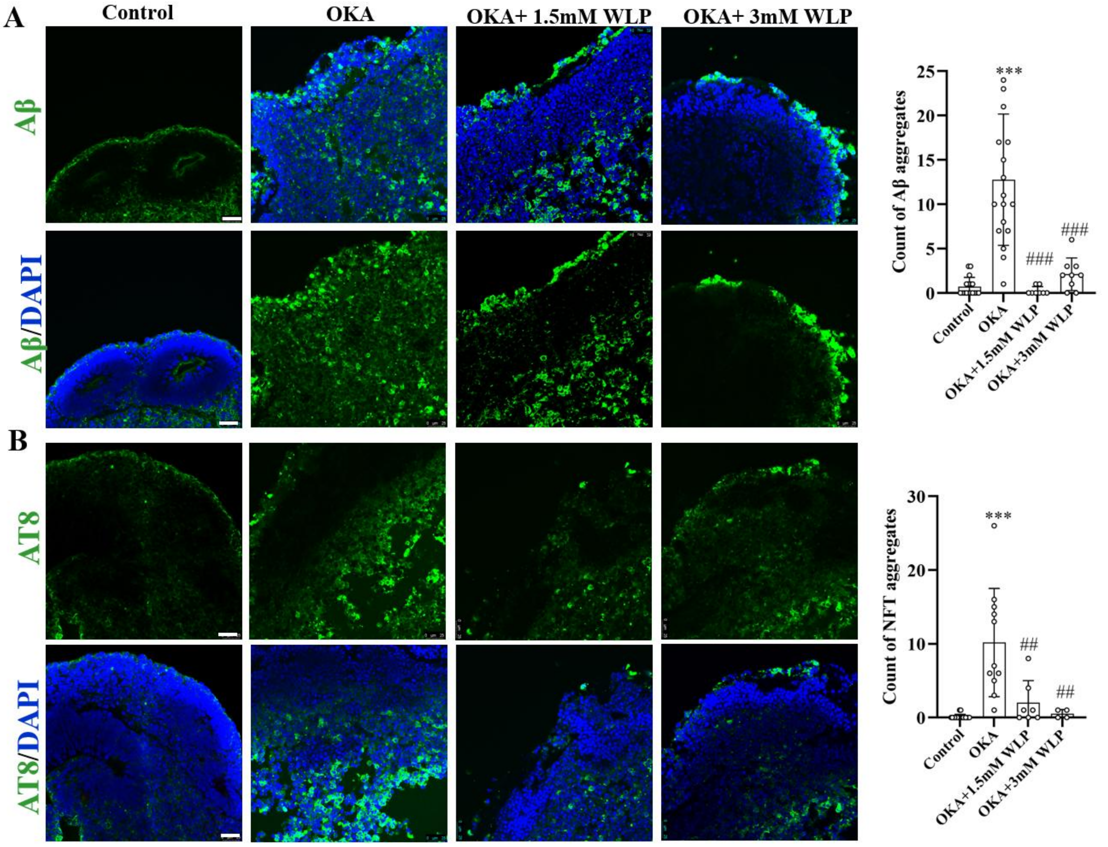

Figure S1. Protective effect of 1.5-3.0 mM WLP peptide on OKA-induced AD pathologies in cerebral organoids (A-B) Representative immunostaining images of A*β* and AT8 (a marker of phosphorylated tau) in control, OKA-treated, OKA + 1.5 mM-treated, and OKA + 3.0 WLP-treated cerebral organoids. Scale bars, 25 μm. Results are presented as mean ± SD (n = 6). Statistical significance was assessed using one-way ANOVA, followed by post-hoc analysis, with *P*-values denoted as ****P* $<$0.001, ##*P*$<$0.01, ###*P*$<$0.01. * represents the statistical significance compared to the control group, while # denotes the statistical significance compared to the OKA-treated group.

Table S1

Antibodies used in study.

Antibody	Source	Dilution	Supplier	Catalog Number
SOX2	Rabbit	1:200	Cell signaling	3579
TUJ1	Mouse	1:1000	Cell signaling	86298
Ki67	Rabbit	1:1000	Cell signaling	ab15580
SYN1	Rabbit	1:400	Cell Signaling	No. 5297
Aβ	Rabbit	1:200	Cell signaling	8243
PSD95	Mouse	1:200	Invitrogen	No. 12605-010
AT8	Rabbit	1:100	Invitrogen	MN1020
DAPI	/	1:1000	VECTOR	H-1200
Tunel	/	/	Roche	12156792910

Table S2. Differential gene expression among control, OKA, and WLP groups (Gene Set).

Gene name	Control vs OKA			OKA vs WLP		
	Log$_2$FC	*P* value	Regulate	Log$_2$FC	*P* value	Regulate
MIAT	-3.27	0	down	0.39	0.002883	up
MIR9-3HG	-3.24	0	down	0.31	0.011665	up
GABPB1-AS1	-3.27	3.19E-246	down	0.32	0.029425	up
AGO1	-2.58	5.17E-162	down	0.33	0.009378	up
NUP210	-3.06	9.79E-144	down	0.39	0.014663	up
CRHR2	-4.48	2.02E-138	down	0.68	0.029713	up
FAR2P4	-2.10	5.42E-126	down	0.37	0.000525	up
FAT3	-1.73	1.65E-113	down	0.32	0.001751	up
SLC35E2B	-1.96	4.45E-112	down	0.43	0.000373	up
H6PD	-2.85	6.11E-111	down	0.56	0.005093	up
RIMS3	-1.93	2.94E-104	down	0.34	0.011919	up
KCNQ3	-2.10	3.05E-81	down	0.29	0.043385	up
TMTC1	-2.14	1.44E-76	down	0.32	0.031986	up
CHD3	-1.10	1.94E-76	down	0.40	0.001216	up
MARCHF4	-2.34	2.83E-76	down	0.47	0.033563	up
DNM1	-1.66	1.18E-71	down	0.34	0.004381	up
ZBTB37	-1.73	2.07E-67	down	0.30	0.008999	up
NPEPL1	-2.73	1.37E-65	down	0.49	0.039375	up
ZNF718	-1.46	1.81E-64	down	0.26	0.007777	up
PLCXD1	-1.68	5.39E-63	down	0.28	0.025024	up
KCTD15	-2.18	7.76E-63	down	0.43	0.013323	up
CD101	-3.47	2.38E-60	down	1.23	0.003669	up
GOLGA8A	-1.25	1.07E-59	down	0.28	8.72E-05	up
IL3RA	-1.88	2.71E-51	down	0.58	9.68E-06	up
AKT2	-1.29	1.46E-49	down	0.28	0.000473	up
EXOG	-1.42	3.58E-49	down	0.36	0.001699	up
ENSG00000237094	-1.50	2.80E-47	down	0.41	0.000676	up
JMJD7-PLA2G4B	-2.30	4.49E-45	down	0.39	0.019675	up
KCNIP2	-2.54	6.43E-45	down	0.62	0.021311	up
ENSG00000286909	-1.84	1.45E-39	down	0.38	0.037868	up
ZNF528	-1.29	2.89E-39	down	0.38	0.003377	up
SLCO5A1	-1.45	3.63E-39	down	0.38	0.004739	up
GRIN1	-1.69	2.48E-38	down	0.47	0.004324	up
ARMCX4	-1.42	4.49E-37	down	0.44	0.002934	up
PASK	-1.31	1.44E-36	down	0.27	0.034262	up
PSMG4	-1.88	1.46E-36	down	0.38	0.048806	up
NSUN5P1	-1.20	4.39E-36	down	0.30	0.002345	up
ENSG00000263809	-1.70	1.46E-35	down	0.40	0.013638	up
PCDHB6	-3.36	2.76E-35	down	0.88	0.048335	up
PABPC1L	-1.52	3.63E-33	down	0.33	0.023366	up
CRYL1	-2.34	3.74E-32	down	0.62	0.023932	up
IDUA	-1.86	7.50E-31	down	0.42	0.04094	up
ZNF772	-1.50	9.19E-30	down	0.39	0.022731	up
PLXNC1	-1.48	1.95E-29	down	0.38	0.023123	up
HCN3	-1.38	2.30E-29	down	0.58	4.86E-05	up
ENSG00000273151	-1.28	3.19E-27	down	0.50	0.000292	up
ZNF160	-0.98	2.03E-25	down	0.31	0.004002	up
PPP1R3E	-1.39	2.98E-25	down	0.36	0.028353	up
GPR173	-1.00	6.28E-25	down	0.37	0.000615	up
SIRT3	-1.34	1.28E-24	down	0.47	0.00264	up
ENSG00000254929	-0.97	1.29E-24	down	0.45	0.000314	up

Gene name	Control vs OKA			OKA vs WLP		
	Log_2FC	*P* value	Regulate	Log_2FC	*P* value	Regulate
LINC00342	-0.80	2.71E-24	down	0.33	0.000297	up
ENSG00000287490	-1.61	3.11E-24	down	0.42	0.021956	up
RPL32P3	-0.99	4.61E-23	down	0.37	0.001254	up
PIK3R2	-1.92	1.10E-22	down	0.72	9.06E-05	up
YJEFN3	-1.51	2.19E-22	down	0.68	0.000275	up
LIMD1	-1.63	1.91E-21	down	0.47	0.027821	up
DIP2A	-0.90	5.16E-21	down	0.31	0.001133	up
MZF1	-1.03	9.22E-21	down	0.53	8.88E-06	up
ZNF320	-0.96	4.06E-20	down	0.32	0.007448	up
TMEM151B	-0.92	1.21E-18	down	0.29	0.024635	up
ANO5	-1.73	1.94E-18	down	0.51	0.044781	up
MCM3AP-AS1	-1.52	5.49E-18	down	0.47	0.045437	up
UVSSA	-0.94	6.32E-18	down	0.31	0.006732	up
ZFHX2	-1.12	8.25E-18	down	0.37	0.03331	up
SV2C	-1.02	1.13E-17	down	0.47	0.000331	up
BMS1P1	-0.99	1.36E-17	down	0.39	0.006564	up
LTO1	-1.04	2.79E-17	down	0.49	0.000506	up
FCHSD1	-1.39	2.82E-17	down	0.72	0.005471	up
ENSG00000284431	-1.63	4.00E-17	down	0.51	0.012798	up
SEMA4D	-0.72	7.39E-17	down	0.30	0.007479	up
ENSG00000242588	-0.91	1.29E-16	down	0.40	2.21E-05	up
DRP2	-1.99	3.99E-16	down	0.72	0.030836	up
ENSG00000272578	-1.38	1.25E-15	down	0.63	0.003369	up
PFN1P6	-1.99	2.20E-15	down	0.66	0.034004	up
HAUS5	-1.01	4.14E-15	down	0.29	0.043771	up
L3MBTL1	-1.33	1.04E-14	down	0.50	0.00788	up
MMP16	-1.00	1.25E-14	down	0.31	0.021149	up
FAM227A	-1.18	1.69E-14	down	0.48	0.016127	up
FTX	-0.83	3.92E-14	down	0.38	0.00637	up
WNT2B	-0.90	4.63E-14	down	0.40	0.002383	up
DNM1P51	-1.11	6.32E-14	down	0.41	0.008602	up
ENTPD4	-0.54	5.23E-13	down	0.31	0.000337	up
PCDHB12	-1.15	2.40E-12	down	0.54	0.004745	up
CICP14	-1.15	2.99E-12	down	0.51	0.004468	up
OSGEP	-0.78	7.67E-12	down	0.27	0.022224	up
NUTM2A	-1.12	1.32E-11	down	0.60	0.002952	up
UBAP1L	-1.28	2.46E-11	down	0.49	0.036256	up
PEX6	-0.94	7.50E-11	down	0.37	0.025817	up
MAP3K4-AS1	-1.93	7.58E-11	down	0.81	0.048037	up
ZNF783	-0.71	9.62E-11	down	0.32	0.006445	up
BSN	-0.62	1.16E-10	down	0.29	0.00488	up
GSDMB	-0.72	1.40E-10	down	0.42	0.000556	up
USP49	-1.02	2.11E-10	down	0.55	0.007076	up
ELK4	-0.77	3.24E-10	down	0.28	0.03282	up
KHNYN	-0.61	3.25E-10	down	0.39	0.000293	up
NCR3LG1	-0.54	3.83E-10	down	0.29	0.00143	up
STARD7-AS1	-1.49	1.75E-09	down	0.76	0.009913	up
MFSD4B	-0.72	2.89E-09	down	0.31	0.035705	up
GOLGA7B	-0.70	4.87E-09	down	0.28	0.035808	up
VAV3	-1.41	5.43E-09	down	0.60	0.034332	up
LINC01002	-0.81	6.38E-09	down	0.32	0.023772	up
ZDHHC20-IT1	-1.56	8.53E-09	down	0.62	0.039981	up

Gene name	Control vs OKA			OKA vs WLP		
	Log_2FC	*P* value	Regulate	Log_2FC	*P* value	Regulate
CLVS2	-1.04	1.44E-08	down	0.54	0.016426	up
CBWD4P	-1.77	2.43E-08	down	0.89	0.020935	up
FAM157C	-0.64	4.21E-08	down	0.30	0.004203	up
NBEAL2	-0.72	6.33E-08	down	0.44	0.012892	up
SMG1P7	-1.50	9.28E-08	down	0.69	0.042749	up
ZNF785	-0.53	1.04E-07	down	0.34	0.001528	up
RASD2	-2.06	1.08E-07	down	1.07	0.021592	up
NRXN3	-0.71	2.42E-07	down	0.41	0.042391	up
CSPG4P10	-0.69	4.35E-07	down	0.32	0.030219	up
SLC24A2	-1.44	4.82E-07	down	0.74	0.023836	up
RGS11	-1.20	7.09E-07	down	0.68	0.028428	up
SCRT2	-0.61	1.32E-06	down	0.32	0.036182	up
MGAT5B	-0.61	1.36E-06	down	0.33	0.019846	up
TUBG2	-0.49	3.42E-06	down	0.35	0.001994	up
C4orf50	-1.38	4.76E-06	down	0.77	0.036852	up
HGSNAT	-0.53	6.23E-06	down	0.30	0.02358	up
ITPR3	-1.56	8.78E-06	down	0.91	0.028997	up
DTYMK	-0.44	9.46E-06	down	0.36	0.002789	up
NMNAT3	-0.60	1.15E-05	down	0.33	0.044948	up
RAB11FIP1P1	-1.89	1.76E-05	down	1.19	0.021531	up
PPT2-EGFL8	-0.55	2.11E-05	down	0.34	0.004925	up
RNF157-AS1	-1.49	3.60E-05	down	1.04	0.028582	up
NFATC2IP	-0.37	0.000109	down	0.31	0.002248	up
TUBGCP6	-0.38	0.0001255	down	0.28	0.004056	up
PALS2	-0.47	0.0003535	down	0.29	0.036727	up
MTG2	-0.61	0.0005661	down	0.43	0.006848	up
ZNF471	-0.40	0.0005774	down	0.42	0.002185	up
FOXP2	-0.56	0.0007967	down	0.50	0.002468	up
KCNC4	-0.45	0.001035	down	0.40	0.00476	up
MSANTD2	-0.34	0.001298	down	0.30	0.009631	up
MIR124-1HG	-0.34	0.001668	down	0.39	0.000846	up
SPATA5	-0.53	0.0019556	down	0.39	0.044664	up
HERC2P7	-0.52	0.0019868	down	0.44	0.024802	up
PCBD2	-0.65	0.0024599	down	0.50	0.036337	up
DLX1	-1.64	0.0025437	down	1.84	0.01659	up
MAP4K2	-0.42	0.0031809	down	0.33	0.038635	up
SHC2	-0.31	0.0032207	down	0.27	0.021528	up
PPP1R12B	-0.32	0.003365	down	0.29	0.013783	up
ZNF814	-0.47	0.0033657	down	0.49	0.008972	up
LINC02538	-3.75	0.0069368	down	3.31	0.029235	up
CHN2	-0.85	0.0084233	down	0.66	0.045007	up
NAIP	-0.31	0.0094146	down	0.31	0.019719	up
FAM149A	-0.43	0.0100407	down	0.40	0.019867	up
TMEM87B	-0.39	0.012906	down	0.38	0.029013	up
PTENP1-AS	-0.34	0.016152	down	0.27	0.049111	up
KCNMB2	-3.41	0.0170137	down	3.15	0.040011	up
SUGT1P4-STRA6LP-CCDC180	-0.34	0.0170462	down	0.35	0.011382	up
LINC00900	-3.40	0.0193672	down	3.11	0.04793	up
ST8SIA5	-0.53	0.0236308	down	0.53	0.038231	up
RPL21P75	-4.75	0.0237745	down	4.43	0.046964	up

Gene name	Control vs OKA			OKA vs WLP		
	Log_2FC	*P* value	Regulate	Log_2FC	*P* value	Regulate
MSL3P1	-0.60	0.0262166	down	0.65	0.008885	up
PKMP3	-3.60	0.026303	down	4.48	0.002666	up

Gene name	Control vs OKA			OKA vs WLP		
	Log_2FC	Pvalue	Regulate	Log_2FC	Pvalue	Regulate
PMAIP1	4.21	2.22E-270	up	-0.42	0.000234486	down
CCN1	3.90	9.04E-253	up	-0.47	1.01001E-07	down
DDIT3	3.42	7.22E-231	up	-0.28	0.005679557	down
GADD45G	3.76	1.17E-199	up	-0.32	0.007150477	down
TNFSF9	5.67	7.23E-172	up	-0.37	0.00351023	down
BAG3	3.27	3.79E-166	up	-0.34	0.000491807	down
HMOX1	4.36	1.99E-162	up	-0.75	4.94533E-11	down
ZFP36	4.70	5.45E-155	up	-0.43	0.000789193	down
TNFRSF12A	4.19	1.82E-147	up	-0.29	0.019401421	down
THBS1	4.54	2.93E-147	up	-0.44	8.00126E-05	down
ADM	2.80	7.56E-141	up	-0.73	1.42241E-13	down
SERPINE1	5.00	1.89E-137	up	-0.35	0.011407773	down
DIO3	4.60	1.34E-121	up	-0.67	4.67783E-05	down
SOCS3	4.76	1.57E-121	up	-0.33	0.027925231	down
TENT5A	4.77	6.24E-120	up	-0.59	8.20017E-05	down
H2BC12	3.34	2.61E-112	up	-0.36	0.008181688	down
SNHG15	2.11	3.04E-110	up	-0.34	0.000209799	down
GADD45B	6.44	8.86E-107	up	-0.41	1.63852E-05	down
SNHG1	1.72	1.79E-106	up	-0.30	1.76347E-05	down
RPL13AP5	3.43	4.19E-96	up	-0.30	0.013197824	down
FTL	2.19	2.22E-92	up	-0.35	0.000191312	down
GEM	2.52	4.29E-91	up	-0.31	0.004542449	down
TRMT10C	2.17	7.05E-90	up	-0.33	0.001134454	down
TBC1D22A-DT	2.62	2.50E-88	up	-0.37	0.000389855	down
FAM166A	5.28	4.00E-88	up	-0.39	0.019903842	down
H2BC11	3.38	9.04E-88	up	-0.37	0.009604313	down
H2BC8	3.14	1.01E-73	up	-0.46	0.000871694	down
CCN2	3.05	4.14E-73	up	-0.57	7.51693E-05	down
SMU1	1.76	2.45E-72	up	-0.39	9.8564E-05	down
ATP6V1G1	1.21	1.23E-70	up	-0.27	0.000344487	down
VASP	2.09	2.20E-68	up	-0.33	0.004360449	down
RNU5A-1	6.66	2.32E-68	up	-0.29	0.0474643	down
HSPA1A	3.65	3.68E-68	up	-0.79	2.64069E-16	down
EN2	4.87	3.57E-67	up	-0.32	0.037661963	down
ANGPT2	3.49	5.43E-65	up	-0.48	0.029729722	down
KLHL41	5.56	6.59E-58	up	-1.19	0.020571477	down
RRAD	5.38	1.29E-53	up	-0.39	0.001451799	down
H1-2	3.14	2.47E-52	up	-0.64	0.000432557	down
ICAM1	4.13	2.30E-49	up	-1.16	7.7743E-07	down
OVOL1	6.10	2.73E-49	up	-0.54	0.00504724	down
CKS2	1.52	1.72E-48	up	-0.27	0.00235318	down
RNU5D-1	6.76	4.96E-46	up	-0.32	0.042533048	down
NFATC1	2.70	1.18E-44	up	-0.31	0.041834872	down
TCEAL9	1.41	1.36E-44	up	-0.37	0.000273326	down
ZNF830	1.67	1.70E-44	up	-0.28	0.010858099	down
CCDC80	2.67	1.02E-43	up	-1.03	0.004688585	down
CRABP2	1.17	1.34E-43	up	-0.41	6.41115E-06	down
LINC01962	4.33	4.97E-41	up	-0.51	0.013687486	down
H3C12	5.12	9.34E-40	up	-0.91	0.000218547	down
MT-RNR1	1.03	1.53E-38	up	-0.35	1.0884E-05	down

Gene name	Control vs OKA			OKA vs WLP		
	Log_2FC	Pvalue	Regulate	Log_2FC	Pvalue	Regulate
ARRDC2	3.00	1.41E-36	up	-0.38	0.039667562	down
IER5	1.37	4.19E-35	up	-0.38	0.00018767	down
CDK2AP2	1.89	1.03E-34	up	-0.35	0.004478871	down
CA2	1.51	5.49E-34	up	-0.34	0.000543145	down
DEPP1	2.42	6.01E-34	up	-0.54	0.001755949	down
SLC9A3R1	1.87	2.50E-33	up	-0.46	0.00227746	down
NFKB2	1.67	2.83E-33	up	-0.29	0.017843202	down
C1orf35	1.22	3.19E-33	up	-0.35	0.002582266	down
ID1	1.94	5.07E-32	up	-0.65	3.12252E-05	down
HSPA2	3.31	2.94E-31	up	-0.79	0.000649078	down
TENT5B	4.29	2.98E-31	up	-0.67	0.004204406	down
ATF5	1.07	1.21E-30	up	-0.27	0.005267778	down
H3C10	3.93	1.41E-30	up	-0.87	0.000462706	down
KRT8	1.42	2.18E-30	up	-0.92	2.09318E-09	down
HSPA6	4.29	4.51E-30	up	-0.92	1.88359E-15	down
GATA2	4.65	4.53E-30	up	-0.68	0.011126401	down
CXCL14	2.49	1.63E-29	up	-0.37	0.031591978	down
IER3-AS1	4.67	2.68E-29	up	-0.93	0.001293703	down
EDN1	4.49	6.25E-29	up	-0.47	0.039368263	down
FOXQ1	4.42	2.09E-28	up	-0.57	0.031962965	down
H2BC18	2.06	1.50E-26	up	-0.61	0.000234838	down
CCDC144NL	1.61	3.03E-26	up	-0.49	0.000903927	down
KRT18	1.89	3.06E-26	up	-0.75	1.24316E-06	down
NQO1	1.70	1.66E-25	up	-0.60	0.001432638	down
CXCL8	12.33	1.99E-25	up	-0.31	0.030938817	down
SRSF2	1.15	1.61E-24	up	-0.32	0.001324597	down
TCEAL7	1.35	2.07E-24	up	-0.35	0.003450024	down
FOLR3	5.31	2.20E-24	up	-0.68	0.015601919	down
KRT17	4.26	8.72E-24	up	-0.84	0.00155335	down
RNVU1-31	11.75	3.66E-23	up	-0.68	9.15265E-09	down
H4C14	2.38	5.13E-22	up	-0.29	0.047613352	down
HSPB8	3.27	1.20E-21	up	-0.74	0.004668176	down
EPHA2	1.06	2.18E-21	up	-0.32	0.002661779	down
SNHG8	1.32	4.05E-21	up	-0.40	0.001777495	down
DKK1	4.98	4.50E-21	up	-0.62	0.042018138	down
CXCR4	0.81	9.19E-21	up	-0.57	3.51356E-08	down
ZACN	3.52	1.51E-20	up	-1.38	2.56131E-06	down
EGR2	9.61	1.93E-20	up	-0.52	0.001410495	down
RN7SK	3.36	2.77E-20	up	-1.11	0.000250403	down
TCEAL5	1.35	2.81E-20	up	-0.30	0.036165772	down
IRF7	1.97	7.38E-20	up	-0.73	2.63099E-06	down
GLIPR1	2.20	6.40E-19	up	-0.49	0.020044022	down
SSBL4P	10.65	6.69E-19	up	-0.58	0.004099203	down
NME7	1.11	1.89E-18	up	-0.28	0.022869894	down
BRS3	4.11	1.90E-18	up	-1.28	0.002252145	down
KRT6B	10.47	3.85E-18	up	-0.49	0.047203675	down
U1	5.36	8.73E-18	up	-0.84	0.012607623	down
SERTAD3	1.33	2.22E-17	up	-0.32	0.021345023	down
FOXC1	3.13	3.78E-17	up	-0.71	0.017824979	down
FRG2	9.97	1.86E-16	up	-0.68	0.00661412	down
KRT16	10.00	2.15E-16	up	-0.77	0.005129241	down
PLAUR	1.52	2.94E-16	up	-0.67	0.001062751	down

Gene name	Control vs OKA			OKA vs WLP		
	Log_2FC	Pvalue	Regulate	Log_2FC	Pvalue	Regulate
PRDM1	3.41	4.57E-16	up	-0.94	0.003809034	down
HBA1	6.49	6.36E-16	up	-1.58	5.90734E-06	down
VASN	1.51	9.67E-16	up	-0.59	0.000975867	down
GPNMB	1.66	1.30E-15	up	-0.58	0.014267454	down
GALNTL6	1.57	1.37E-15	up	-0.53	0.006041052	down
POLG-DT	4.14	1.55E-15	up	-1.44	6.417E-05	down
THBD	3.62	1.96E-15	up	-0.70	0.039011923	down
MIR23AHG	1.54	3.71E-15	up	-0.38	0.027959932	down
AFMID	1.27	2.08E-14	up	-0.34	0.023461203	down
LSR	1.86	3.77E-14	up	-0.65	0.016935288	down
LINC02457	4.92	1.05E-13	up	-1.21	0.001918344	down
FRG2B	9.11	1.55E-13	up	-0.69	0.028975064	down
TIMP3	0.82	1.98E-13	up	-0.34	0.029654235	down
MYL12A	1.00	5.95E-13	up	-0.34	0.043319682	down
CALB1	0.78	9.53E-13	up	-0.53	0.000127951	down
ATOH1	5.67	9.62E-13	up	-1.17	0.004755757	down
SFN	5.84	2.84E-12	up	-0.91	0.019751504	down
EDARADD	3.62	3.06E-12	up	-1.83	4.86791E-05	down
RN7SL1	0.93	4.06E-12	up	-0.65	5.26273E-07	down
CLIC1	0.62	1.25E-11	up	-0.29	0.003161229	down
SNHG19	1.44	2.11E-11	up	-0.51	0.032529082	down
NDUFC2-KCTD14	0.90	2.80E-11	up	-0.34	0.009687054	down
KRTAP19-5	8.32	3.06E-11	up	-0.86	0.03038473	down
FRG2C	8.16	4.30E-11	up	-0.72	0.032706062	down
PTCRA	5.18	5.58E-11	up	-2.99	4.09319E-06	down
EPAS1	1.34	5.61E-11	up	-0.56	0.002627247	down
HLA-B	2.31	7.16E-11	up	-0.36	0.005597461	down
S100A11	2.16	9.30E-11	up	-0.72	0.003209872	down
FSCN3	3.05	1.07E-10	up	-0.80	0.033993844	down
NR4A2	1.02	1.16E-10	up	-0.49	0.001262637	down
IFIT2	2.84	1.29E-10	up	-0.65	0.036145317	down
HSPB1	0.74	1.42E-10	up	-0.76	1.30221E-15	down
TAGLN	1.42	3.99E-10	up	-0.41	0.032492316	down
RPL21P11	1.73	4.01E-10	up	-0.49	0.037000478	down
GGNBP1	5.42	4.08E-10	up	-1.30	0.005448335	down
MSX1	1.73	7.43E-10	up	-0.85	0.002344097	down
TAGLN2	0.79	8.59E-10	up	-0.38	0.019182505	down
COA6-AS1	1.99	1.03E-09	up	-0.62	0.031204487	down
LINC00923	1.94	1.34E-09	up	-0.84	0.008134706	down
TRMT10A	1.20	1.49E-09	up	-0.48	0.012144162	down
GPR1-AS	3.90	2.45E-09	up	-1.47	0.005433275	down
CDK13-DT	2.60	2.59E-09	up	-0.75	0.044952408	down
LINC00698	1.80	5.92E-09	up	-0.68	0.032121731	down
H2AJ	1.17	6.06E-09	up	-0.69	0.000560318	down
H2AC12	4.91	7.82E-09	up	-1.41	0.006134998	down
POU4F1	2.43	1.52E-08	up	-0.89	0.016686067	down
TSPEAR	2.79	2.07E-08	up	-1.25	0.007836628	down
CCDC63	7.46	2.63E-08	up	-1.89	0.003695519	down
BAIAP2L1	1.22	2.81E-08	up	-0.50	0.022840377	down
LINC01635	4.48	3.01E-08	up	-2.31	0.000148194	down
PSMG1	0.81	3.48E-08	up	-0.30	0.032179901	down

Gene name	Control vs OKA			OKA vs WLP		
	Log_2FC	Pvalue	Regulate	Log_2FC	Pvalue	Regulate
TFAP2A-AS2	2.86	5.00E-08	up	-0.93	0.02342438	down
CLU	0.39	5.28E-08	up	-0.30	5.6149E-05	down
ELF3	2.75	5.79E-08	up	-0.81	0.048048005	down
LINC01239	2.75	7.12E-08	up	-0.99	0.023177309	down
CPN1	6.33	1.41E-07	up	-2.20	0.000768137	down
CCDC74A	0.85	3.79E-07	up	-1.60	3.75924E-19	down
ARL2-SNX15	1.50	4.06E-07	up	-0.82	0.009972436	down
GPX1	0.50	4.46E-07	up	-0.27	0.031082979	down
TCL6	3.67	5.35E-07	up	-2.00	0.001157693	down
C2orf16	1.02	8.58E-07	up	-0.40	0.02603926	down
LINC02356	3.99	1.15E-06	up	-2.53	0.000149425	down
EML3	0.79	1.27E-06	up	-0.40	0.006393568	down
XIRP1	5.16	1.35E-06	up	-1.32	0.037464129	down
LINC01926	6.88	1.59E-06	up	-1.77	0.030465644	down
ANKRD1	3.56	2.00E-06	up	-1.31	0.017957951	down
LINC01476	4.36	2.01E-06	up	-2.82	0.000244628	down
NEURL1-AS1	6.63	2.24E-06	up	-2.42	0.003529406	down
MINCR	1.31	2.29E-06	up	-0.55	0.036514552	down
PPP1R10	0.41	3.61E-06	up	-0.31	0.001217632	down
GNG12	0.42	4.44E-06	up	-0.43	0.006774335	down
ZUP1	0.64	5.59E-06	up	-0.35	0.019304818	down
MRPL4	0.61	6.73E-06	up	-0.30	0.01778426	down
ZNF37A	1.22	7.06E-06	up	-0.76	0.00413048	down
HNF1A-AS1	2.63	7.40E-06	up	-1.35	0.015194833	down
EGLN3	0.56	9.69E-06	up	-0.36	0.007095089	down
H1-4	4.26	1.06E-05	up	-3.07	0.000593929	down
TACSTD2	6.08	1.14E-05	up	-2.07	0.006893143	down
ANKRD37	0.94	1.50E-05	up	-0.41	0.030041771	down
CAVIN1	0.85	1.88E-05	up	-0.74	0.000362941	down
SDC4	0.65	2.01E-05	up	-0.48	0.012303266	down
DYNLT2	0.88	4.27E-05	up	-0.49	0.022892781	down
PPL	0.84	5.85E-05	up	-0.52	0.011945143	down
MSX2	1.30	6.12E-05	up	-1.26	0.000247667	down
H2BU1	0.86	6.34E-05	up	-0.74	0.000941551	down
CRYAB	2.71	6.76E-05	up	-1.17	0.000604115	down
DCTN6-DT	4.12	7.63E-05	up	-1.68	0.024352284	down
SAT1	0.33	7.93E-05	up	-0.32	0.001723118	down
DGKZP1	6.10	8.71E-05	up	-4.60	0.002721412	down
H2AC18	2.31	0.0001137	up	-1.11	0.03981607	down
PEBP4	6.10	0.0001236	up	-4.60	0.003318295	down
IL32	3.12	0.0001336	up	-1.20	0.049374819	down
IQCK	0.50	0.0001523	up	-0.27	0.044370703	down
PKD1L2	1.53	0.0001834	up	-1.25	0.001927661	down
KRT8P50	3.34	0.0001914	up	-1.60	0.02695316	down
AIMP2	0.49	0.0002062	up	-0.43	0.002780216	down
PLA2G1B	3.75	0.0002276	up	-2.48	0.005855161	down
LINC02694	5.33	0.0003417	up	-2.06	0.045174515	down
LINC01618	3.06	0.0003555	up	-0.81	0.000471906	down
LINC00589	5.44	0.0004518	up	-2.47	0.031209717	down

Gene name	Control vs OKA			OKA vs WLP		
	Log_2FC	Pvalue	Regulate	Log_2FC	Pvalue	Regulate
KRTAP19-1	4.66	0.0004676	up	-1.68	0.047257429	down
PEDS1-UBE2V1	0.44	0.0004807	up	-0.44	0.003288184	down
S100A6	1.90	0.0004976	up	-1.10	0.035897855	down
P2RX4	0.77	0.0005014	up	-1.35	9.52976E-08	down
CRYBA4	5.74	0.0005081	up	-3.33	0.023145592	down
COMT	1.27	0.0006237	up	-0.78	0.029915535	down
AFP	7.14	0.0006485	up	-5.63	0.00737787	down
CHRNB3	4.16	0.0006755	up	-2.40	0.015608133	down
CUBNP2	4.52	0.0008373	up	-2.20	0.023069302	down
LINC01500	3.15	0.0010385	up	-3.19	0.002559101	down
FAM138F	5.43	0.0018499	up	-3.93	0.020806025	down
POP4	0.51	0.0022173	up	-0.32	0.04258524	down
CFAP97D2	2.60	0.0023229	up	-2.84	0.002046176	down
RXFP1	3.42	0.0032174	up	-2.53	0.023177426	down
SEC14L6	1.51	0.0034509	up	-1.79	0.001986735	down
MLC1	0.44	0.0036577	up	-0.65	4.77376E-05	down
SERPINA3	1.25	0.0039704	up	-1.05	0.017394988	down
TANK-AS1	3.79	0.0040926	up	-4.73	0.002244483	down
SH3TC1	1.59	0.004243	up	-2.85	4.40389E-06	down
LINC01892	2.93	0.0044279	up	-1.53	0.036801342	down
H2BC21	0.32	0.0048863	up	-0.84	8.94008E-10	down
FGF7P3	0.80	0.0050289	up	-0.59	0.047404444	down
LDHC	2.69	0.0054195	up	-1.97	0.035373078	down
TRPC2	5.21	0.0054767	up	-3.71	0.0408777	down
LINC01220	4.47	0.0087021	up	-3.06	0.049928555	down
HBZ	2.32	0.0105559	up	-2.19	0.023271	down
CLDN4	0.73	0.0125709	up	-0.71	0.009273692	down
AARSD1P1	5.14	0.0136157	up	-4.60	0.02928089	down
H2AC16	2.53	0.0166158	up	-2.34	0.039622581	down
LINC01998	4.78	0.0214847	up	-4.24	0.043895015	down
AGA-DT	1.85	0.0228962	up	-1.56	0.038032904	down
VPS33B	0.38	0.0229357	up	-0.49	0.025974614	down
USP17L15	4.91	0.0245291	up	-4.37	0.048377304	down
NPM3	0.39	0.0280258	up	-0.38	0.045464274	down
ACTC1	2.86	0.0294782	up	-1.55	0.008783303	down
H2AW	0.46	0.0296942	up	-0.54	0.002924961	down
PROSER2-AS1	2.20	0.030428	up	-3.64	0.009211992	down
SPICE1	0.40	0.0335185	up	-0.42	0.043577754	down
ZNF503	0.81	0.0392987	up	-0.67	0.003750948	down
TRPM6	1.37	0.0399829	up	-1.35	0.048109718	down
C1orf167	2.09	0.0420451	up	-3.14	0.01251407	down
